# Long-term efficacy and feasibility of levonorgestrel-releasing intrauterine device use in patients with adenomyosis

**DOI:** 10.1097/MD.0000000000020421

**Published:** 2020-05-29

**Authors:** Soo Youn Song, Sun Yeul Lee, Hye Yun Kim, Dan Bit Park, Da Eun Kim, Ki Hwan Lee, Siyeo Lee, Jung Bo Yang, Heon Jong Yoo

**Affiliations:** a Department of Obstetrics and Gynecology; b Department of Anesthesiology and Pain Medicine, Chungnam National University School of Medicine; c Department of Biochemistry, College of Natural Sciences, Chungnam National University, Daejeon, Republic of Korea.

**Keywords:** adenomyosis, dysmenorrhea, levonorgestrel-releasing intrauterine device, menorrhagia

## Abstract

To evaluate the efficacy and feasibility of levonorgestrel-releasing intrauterine device (LNG-IUD) use longer than 5 years in women with adenomyosis.

Data were retrospectively collected from patients who were treated with LNG-IUD longer than 5 years at the Chungnam National University hospital for adenomyosis diagnosed with ultrasonography from January 2006 to November 2013.

A total of 131 patients who were diagnosed with adenomyosis had treated with LNG-IUD longer than 5 years. The mean duration of keeping 1 device without replacement was 58.35 ± 15.98 months, and total duration of LNG-IUD treatment was 83.86 ± 23.88 months. A total of 51 patients stopped using LNG-IUD after 5 years and the mean age at the time of LNG-IUD removal was 52.46 ± 6.9. LNG-IUD treatment had a significant effect on both menorrhagia and dysmenorrhea starting from the first month of insertion (*P* < .01), which persisted until 6 years when the effect started to plateau. The decrease in uterine volume was not consistent during the treatment period. The uterine volume decreased significantly only in the first and second year of LNG-IUD treatment and then from eighth to tenth year of LNG-IUD treatment (*P* < .05). Adverse events after insertion of LNG-IUD decreased significantly after 5 years.

LNG-IUD treatment longer than 5 years is an effective and feasible method for patients diagnosed with adenomyosis.

## 1 Introduction

Adenomyosis is a common benign gynecologic disorder that is characterized by heterotropic endometrial glands and stroma in the myometrium.^[1]^ Although the exact incidence of adenomyosis diagnosed histopathologically is hard to predict, it is believed that about 20% of women in reproductive age suffer from the disease.^[2]^ About two-thirds of women who are diagnosed with adenomyosis are symptomatic and the most common symptoms include menorrhagia and dysmenorrhea.^[3,4]^ Other symptoms include enlarged, tender uterus and chronic pelvic pain.^[5,6]^

Hysterectomy has been traditionally used as the only definitive curative treatment option. More recently, other medical treatments using suppressive hormonal treatment, such as oral contraceptives, danazol, aromatase inhibitor, gonadotropin releasing hormone analog have been used to control symptoms of adenomyosis among women who were unwilling to undergo hysterectomy.^[7]^

The levonorgestrel-releasing intrauterine device (LNG-IUD), which releases 20 μg of levonorgestrel every 24 hours during a 5-year period, has been proven to be effective for menorrhagia and dysmenorrhea.^[8,9]^ Studies have revealed that LNG-IUD effectively reduced the pain and the uterine volume in women with adenomyosis.^[10,11]^ Although LNG-IUD is used widely among patients in adenomyosis, studies showing the long-term effect of LNG-IUD is limited to 5 years.^[12,13]^ Therefore, in this study we evaluated the efficacy and feasibility of LNG-IUD in women with adenomyosis who used LNG-IUD for more than 5 years.

## 2 Material and methods

This retrospective study was conducted in Chungnam National University hospital and was approved by the Institutional Review Board of the hospital (2019–03–070). Data were collected from patients who underwent LNG-IUD insertion for symptoms such as dysmenorrhea or menorrhagia due to adenomyosis diagnosed with ultrasonography from January 2006 to November 2013. Patients whose last follow-up visit was less than 5 years ago or who stopped using LNG-IUD for less than 5 years were excluded from the analysis. Duration of treatment was defined as sum of total duration of each LNG-IUD device. If there was a time interval longer than 1 week between the removal of the old LNG-IUD and the insertion of the new LNG-IUD, then the duration of keeping the previous LNG-IUD and the duration of keeping the latter LNG-IUD were not integrated.

Adenomyosis was diagnosed based on patients’ symptoms, physical examination and ultrasonography. The diagnostic criteria of ultrasonography were

(1) existence of myometrial cyst,
(2) heterogenous myometrial echotexture or distortion of myometrial echotexture,
(3) ill-defined focus of abnormal myometrial echotexture, and
(4) globular and/or asymmetric uterine shape.^[14,15]^

Once adenomyosis was diagnosed, the uterine volume was measured. Uterine length was measured from the fundus to the internal OS of the uterus in the sagittal plane of the vaginal probe. The anteroposterior and transverse diameter was measured in a transverse plane to visualize the maximum anteroposterior diameter at the uterine corpus. The uterine volume was calculated by multiplying all the 3 measures with a constant 0.52 (length × anteroposterior diameter × transverse diameter × 0.52).^[12]^

Demographic data, characteristics of menstruation, obstetrical, medical and surgical history were retrieved from the medical records. Blood loss was estimated using a pictorial blood assessment chart (PBAC) suggested by Higham,^[16]^ and pain was measured on a 10 cm visual analog scale (VAS).^[17]^ A PBAC score >150, which is equivalent to more than 80 mL of blood loss was defined as menorrhagia.^[18]^ After initial screening, patients were followed up at 3, 6, 9, and 12 months during the first year after LNG-IUD insertion, followed by annual visits thereafter.

Adverse effects of LNG-IUD were defined as symptoms and signs of the patients that the physician have mentioned in the medical records as pertinent events related to the LNG-IUD treatment. Adverse events included irregular bleeding, abdomen discomfort, ovarian cyst, breast discomfort, leukorrhea and itching. Treatment failure was defined as a removal of LNG-IUD due to persistent or aggravated symptoms and conversion to other types of treatment such as hormone therapy or hysterectomy.

SPSS version 24.0 (IBM, Armonk, NY) was used for statistical analysis. Changes in PBAC, VAS or uterine volume were measured using a paired sample *t*-test when the data were evenly distributed, otherwise Wilcoxon signed rank test was used. A *P* value less than .05 was considered significant.

## 3 Results

**Figure 1:**
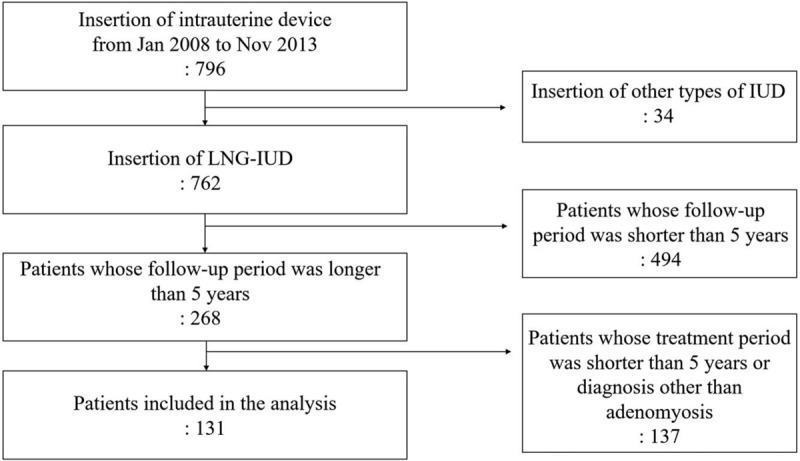
Flow chart outlining the selection of patients. IUD = intrauterine device, LNG-IUD = levonorgestrel-releasing intrauterine device.

From January 2008 to November 2011, 796 patients underwent LNG-IUD insertion (Fig. 1). Among those who underwent the treatment, 268 patients had a follow-up period longer than 5 years. Excluding patients who were not diagnosed with adenomyosis, a total of 131 patients had LNG-IUD in situ for longer than 5 years.

Table 1 presents the characteristics of patients. The mean age at the time of first LNG-IUD insertion was 42.07 ± 4.17, and the mean age at the time of first LNG-IUD replacement was 47.27 ± 4.33. The mean duration of total follow-up period was 88.97 ± 25.27 months. The mean duration of keeping 1 device without replacement was 58.35 ± 15.98 months, and total duration of LNG-IUD treatment was 83.86 ± 23.88. months. A total of 51 patients stopped using LNG-IUD after 5 years and the mean age at the time of LNG-IUD removal was 52.46 ± 6.9. A majority of the patients (27/51) stopped using LNG-IUD because of menopause. Ten patients underwent LNG-IUD removal for loss of pain and menorrhagia. Among those 10 patients, only 1 patient complained of relapse of menorrhagia and dysmenorrhea 1 month after removal and was treated with oral contraceptive. The reasons for the removal of LNG-IUD by the remaining 14 patients were irregular bleeding, increased uterine volume, self-expulsion, persistent symptoms, abdominal discomfort, coital discomfort, and leukorrhea. Seventy-two patients manifested concurrent leiomyoma, and 17 patients had concurrent endometriosis.

**Table 1:**
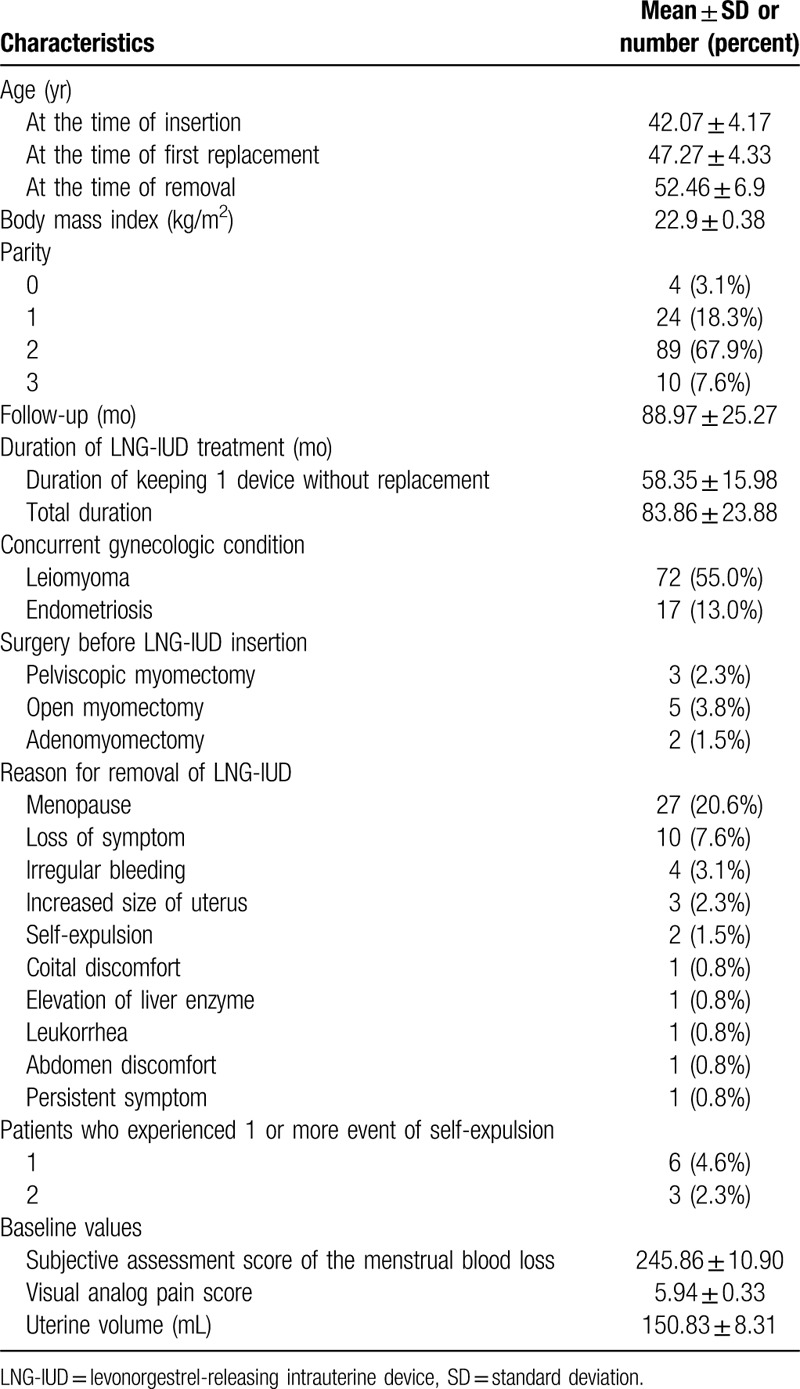
Baseline and clinical characteristics of patients.

Although most women changed the device around 58 months, there were a few women who changed the device earlier or later. The number of patients who changed the first device in less than 5 years was 18. Among those, 9 of them changed the device after 4 years, 5 patients after 2 years, 3 patients after 1 year, and 1 patient at same year. 9 out of 18 patients replaced the device due to self-expulsion. The number of patients who changed the second device in less than 5 years were 4. Three patients were the same patient who experienced self-expulsion with first device, and they reinserted the device after second self-expulsion. One patient replaced the device due to increased amount of vaginal bleeding. The number of patients who retained first device more than 5 years was 6. Except for 1 patient who removed the device after 6 years, remaining 5 patients did not visit the hospital regularly, and forgot to remove the device.

Five patients out of 131 patients (3.8%) underwent hysterectomy after 5 years including 2 for dysmenorrhea and menorrhagia despite more than 5 years’ use of LNG-IUD, 2 for increased uterine volume after 5 years of LNG-IUD treatment, 1 for dysmenorrhea and menorrhagia after self-expulsion of LNG-IUD.

**Figure 2:**
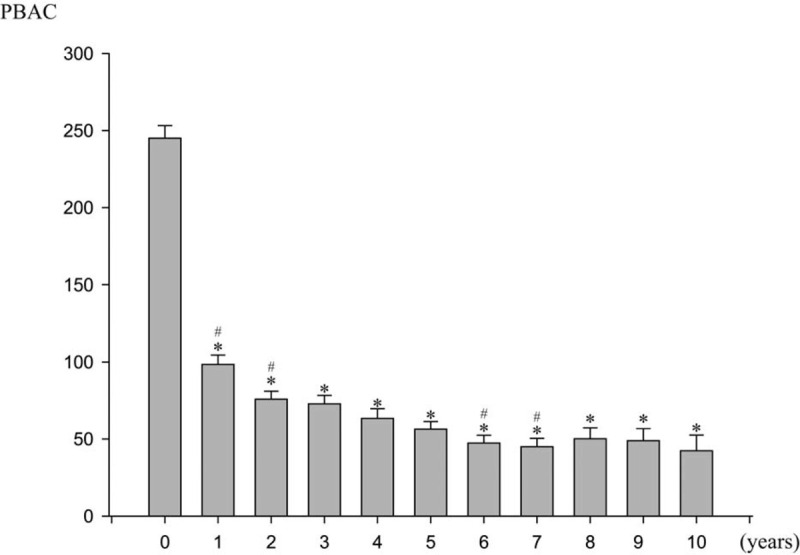
Changes in subjective assessment score of menstrual blood loss during 10 yr of LNG-IUD treatment. LNG-IUD = levonorgestrel-releasing intrauterine device. ^∗^ significant difference compared with the basline value, *P* < .05. ^#^ significant difference compared with the value of previous month, *P* < .05.

Figure 2 illustrates the changes in PBAC over a 10-year period. The mean PBAC before LNG-IUD insertion was 245.11 (range 25–300). The mean PBAC decreased to 56.40 after 5 years of treatment (*P* < .001), and to 42.37 after 10 years of treatment (*P* < .001). Decrease of PBAC was most prominent in the first year. Although the mean PBAC score continued to decrease until the sixth year, the annual difference was small. After 6 years, the mean PBAC score nearly plateaued.

**Figure 3:**
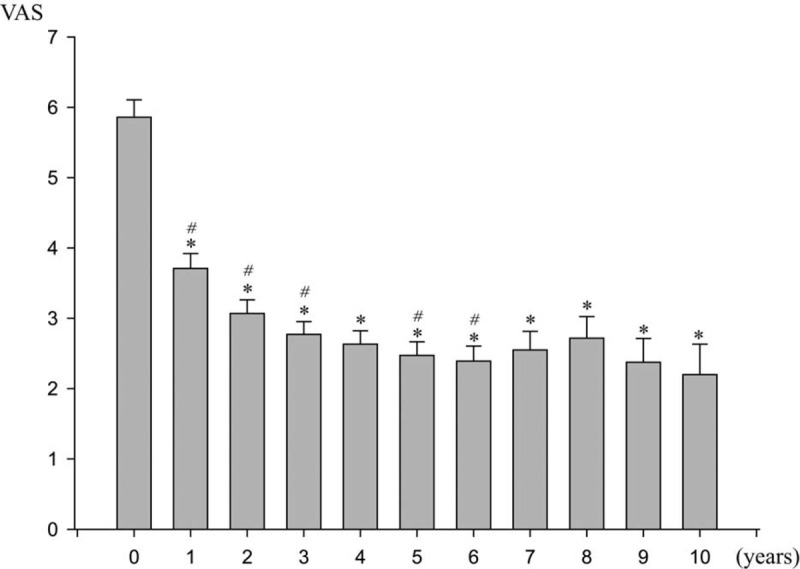
Change in visual analog pain scale during 10 yr of LNG-IUD treatment. LNG-IUD = levonorgestrel-releasing intrauterine device. ^∗^significant difference compared with the baseline value, *P* < .05. ^#^ significant difference compared with the value of previous month, *P* < .05.

Figure 3 shows the changes in pain score assessed with VAS during the treatment period. The baseline VAS score was 5.86 (range 1–8) before LNG-IUD treatment. The mean VAS decreased to 2.47 after 5 years (*P* < .001), and further to 2.20 after 10 years (*P* < .001). A marked reduction of mean VAS score was observed in the first year of treatment. The degree of pain reduction was small yet consistent over the 6-year period. The mean VAS score after 6 years showed no significant change.

**Figure 4:**
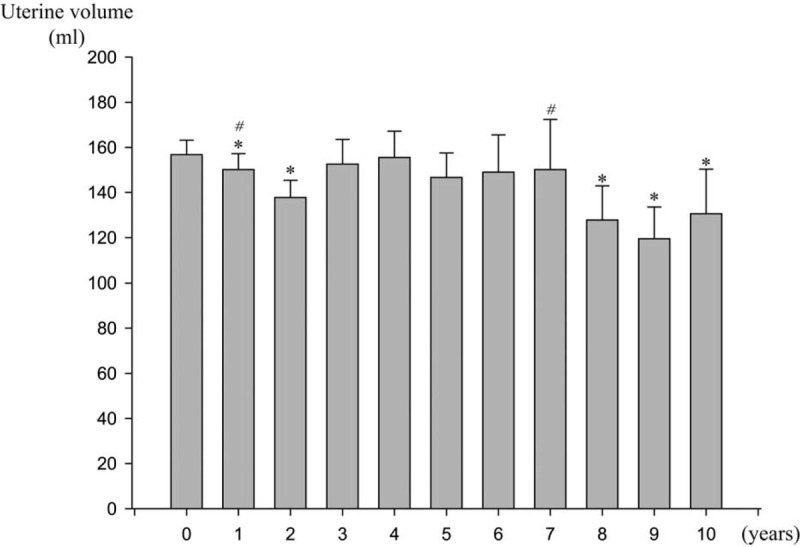
Change in uterine volume during 10 yr of LNG-IUD treatment. LNG-IUD = levonorgestrel-releasing intrauterine device. ^∗^ significant difference compared with the basline value, *P* < .05. ^#^ significant difference compared with the value of previous month, *P* < .05.

As shown in Figure 4, the uterine volume did not show a constant decrease during the treatment period. The initial uterine volume before LNG-IUD insertion was 156.81 ± 73.26 mL. The uterine volume decreased significantly during the first and second years of treatment (*P* = .037, *P* = .006, respectively). However, it started to increase by the third year of treatment. The uterine volume was not significantly decreased from the third to the seventh year compared with the baseline uterine volume. After the seventh year, the uterine volume started to decrease again, and the uterus was significantly smaller in the eighth, ninth and tenth year of treatment compared to the uterus before LNG-IUD insertion (*P* = .006, *P* = .003, *P* = .025, respectively).

**Figure 5:**
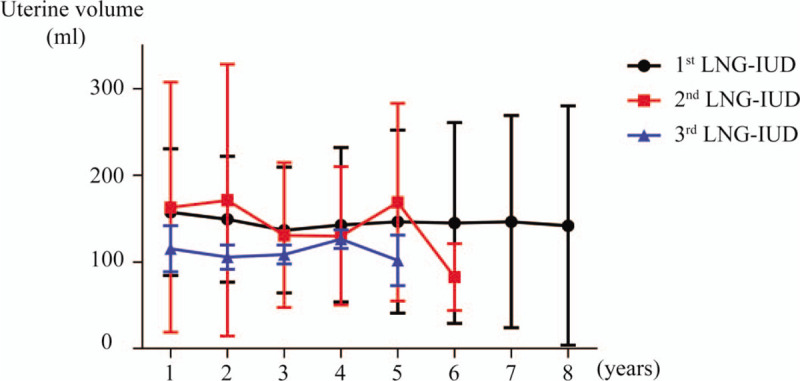
Change in uterine volume grouped by first, second and third LNG-IUD. LNG-IUD = levonorgestrel-releasing intrauterine device.

Figure 5 shows changes in uterine volume grouped as first, second, or third LNG-IUD, because some patients replaced the device once or twice while others retained 1 device for long period of time. Similar to the result shown in Figure 4, uterine volume decreased significantly after 1 and 2 years of LNG-IUD treatment (*P* = .018 and *P* = .006, respectively). However, after 3 years, changes in uterine volume were not significant compared to the baseline value. With second and third LNG-IUD, no significant decrease of uterine volume was observed.

We evaluated the number of patients who experienced adverse events before and after 5 years of LNG-IUD treatment (Table 2). The total number of patients with adverse events was 121 (92.4%) before 5 years and 74 patients had multiple symptoms. The total number of patients with adverse events was 68 (51.9%) after 5 years and 20 patients had multiple symptoms. The total number of patients who had adverse events as well as the number of total adverse events were significantly reduced after 5 years of treatment. During first 5 years, most patients complained of irregular bleeding, leukorrhea, spotting and abdominal discomfort. After 5 years, the frequency of irregular bleeding, spotting as well as leukorrhea were decreased by more than 50%. Itching, headache, breast discomfort, weight gain, and acne were completely resolved after 5 years. However, patients with back pain increased after 5 years of treatment.

**Table 2:**
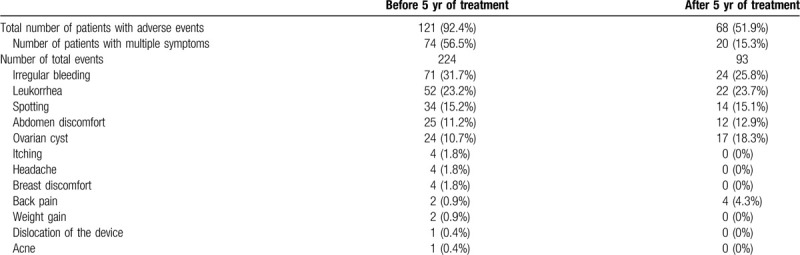
Number of patients who experienced adverse events before and after 5 yr of levonorgestrel-releasing intrauterine device treatment.

## 4 Discussion

In this study, we evaluated the effect and feasibility of LNG-IUD on patients with adenomyosis who were treated with LNG-IUD for more than 5 years. LNG-IUD insertion was significantly effective in alleviating pain and reducing blood loss especially during the first 6 years. Although the effects on pain and blood loss decreased after 6 years of treatment, the pain and blood loss did not increase above baseline value. Effect on uterine volume was not constant throughout the 10-year treatment period. Uterine volume decreased significantly at first and second year of LNG-IUD treatment, but there was no significant difference compared to the baseline after 3 years until eighth year. Even if considering the replacement of LNG-IUD, uterine volume decreased after only 1 and 2 years of the first device. Replacement to second or third LNG-IUD did not significantly affect the uterine volume.

The effect of LNG-IUD on pain and blood loss was significant even from the first year. This result was consistent with other studies, which reported dramatic improvement in pain and bleeding pattern as early as 6 months.^[12,19]^ Our study also showed a constant long-term effect of LNG-IUD on pain reduction and decreased the blood loss, which was consistent with the findings of the 3-year study by Sheng.^[13]^ However, the effect on pain and blood loss were minimal after 6 years of treatment. These results are consistent with recent study by Li, which showed that the decrease of pain and blood loss after 2 to 3 years were not statistically significant.^[20]^ Although the pain and blood loss during menstruation remained below baseline throughout 10-year period, 27/131 (20.6%) of women reached menopause after 10 years of treatment in our study. That could have exaggerated the effect of LNG-IUD on the reduction of blood loss and uterine volume. A long-term prospective study with a larger population, controlled for age and menopause is needed to confirm these findings.

The uterine volume after insertion of LNG-IUD decreased significantly compared to baseline after 1 year of treatment, which was consistent with other studies.^[13,19]^ The effect was maintained until the second year, however after 3 years, the uterine size increased to levels comparable to baseline value. Cho also reported a maximal reduction of volume at 12 months, and the recovery of uterine volume at 36 months.^[21]^ On the contrary, Li showed the uterine volume continued to decrease through 5-year period.^[20]^ Our result showed that uterine volume decreased again after 8 years and remained smaller than the baseline value until the 10-year period suggesting the need for long-term treatment of LNG-IUD to observe a significant reduction in uterine volume. Our study also showed that even if LNG-IUD was replaced with new LNG-IUD, the change of uterine volume was not significant compared to the baseline value. This result needs further confirmation with prospective study with larger population.

The incidence of adverse events after LNG-IUD treatment was greatly reduced after 5 years. After 5 years, the incidence of irregular bleeding, leukorrhea, spotting, and abdominal discomfort decreased significantly which is consistent with study by Li, which showed low incidence of adverse events after 5 years of LNG-IUD treatment.^[20]^ Interestingly, unlike other adverse events, the number of patients who complained of back pain doubled from 2 to 4. Increased back pain might be explained by the increased age of subjects. According to a systematic review, the prevalence of chronic back pain increases linearly from 30 years until 60 years of age.^[22]^

Hysterectomy due to symptomatic adenomyosis is not frequent after 5 years of LNG-IUD treatment. Fourteen patients stopped LNG-IUD after 5 years of treatment for symptoms such as irregular bleeding, persistent increase in uterine size or symptoms, coital discomfort, abdominal discomfort, or leukorrhea. Only 5 patients underwent hysterectomy. This result was consistent with the study evaluating the quality of life in patients treated with LNG-IUD over 10 years for menorrhagia, which reported that 90% of women underwent hysterectomy within 5 years of LNG-IUD treatment.^[23]^

This study has several limitations. First, this was a retrospective study with a few missing data from the medical records. Second, adenomyosis was not pathologically confirmed because of the conservative nature of the treatment, which could lead to false-positive or negative diagnosis of adenomyosis. Third, both PBAC and VAS were subjective measures suggesting a risk of over or underestimation. Nonetheless, this study was the first of its kind to evaluate the efficacy and feasibility of LNG-IUD on adenomyosis in patients who were treated with LNG-IUD for more than 5 years.

In conclusion, LNG-IUD treatment for more than 5 years is an effective and feasible method to control pain and blood loss due to adenomyosis, and might be effective in decreasing the uterine volume. Although the effect might not be consistent during the treatment period, subjective scores of pain and blood loss as well as uterine volume did not exceed that of baseline value. Long term treatment with LNG-IUD for more than 5 years is recommended for patients who are unwilling or are contraindicated for surgery.

## Acknowledgments

We thank professor In-Seon Kwon for statistical consults.

## Author contributions

**Conceptualization:** Soo Youn Song, Ki Hwan Lee, Heon Jong Yoo

**Data analysis:** Soo Youn Song, Sun Yeul Lee, Ki Hwan Lee, Jung Bo Yang, Heon Jong Yoo

**Data collection:** Soo Youn Song, Sun Yeul Lee

**Supervision:** Soo Youn Song, Ki Hwan Lee, Heon Jong Yoo

**Writing – original draft:** Soo Youn Song, Sun Yeul Lee, Ki Hwan Lee, Siyeo Lee, Jung Bo Yang, Heon Jong Yoo

**Writing – review & editing:** All authors
